# Ultrasonographic Features of Pleural Thickening in Cats With Pulmonary Carcinoma and Pleural Effusion: A Retrospective Descriptive Study

**DOI:** 10.1002/vms3.71290

**Published:** 2026-10-15

**Authors:** Atsushi Toshima, Caroline V. Fulkerson, Masahiro Murakami

**Affiliations:** ^1^ Japan Small Animal Medical Center Saitama Japan; ^2^ Department of Veterinary Clinical Sciences College of Veterinary Medicine, Purdue University West Lafayette Indiana USA

**Keywords:** cats, lung neoplasms, pleural diseases, pleural effusion, ultrasonography

## Abstract

**Background:**

Pleural involvement is an important but poorly described manifestation of feline pulmonary carcinoma. Thoracic ultrasonography may identify pleural abnormalities and accessible targets for cytologic sampling in cats with pleural effusion.

**Objectives:**

To describe ultrasonographic features of pleural thickening in cats with pulmonary carcinoma and pleural effusion and summarise cytologic findings from pleural effusion and ultrasound‐guided lesion sampling.

**Methods:**

Medical records from one referral hospital were retrospectively reviewed for cats with pulmonary carcinoma or metastatic epithelial neoplasia consistent with pulmonary origin, pleural effusion, and stored thoracic ultrasound images. Images were reviewed in consensus by three observers for distribution, location, shape, maximal thickness, echogenicity, homogeneity, and suspected intercostal muscle invasion.

**Results:**

Twenty‐two cats were included. Pleural thickening was recorded in all cats. Thickening was classified as diffuse in 12 cats, focal in 6, and multifocal in 4; however, scanning was not systematic across all thoracic regions. The representative or measured pleural abnormality was in the cranial pleural region in 18 cats. Maximal pleural thickness was 5.6 ± 3.3 mm (median, 4.9 mm; range, 1.2–16.2 mm). Suspected intercostal muscle invasion was recorded in eight cats. Neoplastic cells were identified in pleural effusion cytology in 15/20 cats. Six cats underwent ultrasound‐guided lesion sampling; cytologic or histopathologic findings were supportive of carcinoma in all 6, including 1 confirmed by biopsy.

**Conclusions:**

In this selected cohort, thoracic ultrasonography identified pleural thickening with variable morphology. Thickened pleura may represent a feasible sampling target, but prospective studies are needed to determine prevalence, distribution, sensitivity, and specificity.

## Graphical abstract

In cats with pulmonary carcinoma and pleural effusion, thoracic ultrasonography identified pleural thickening with variable morphology. In selected cats, thickened pleura or pleura‐associated lesions provided accessible targets for ultrasound‐guided sampling, supporting their potential role in diagnosis.

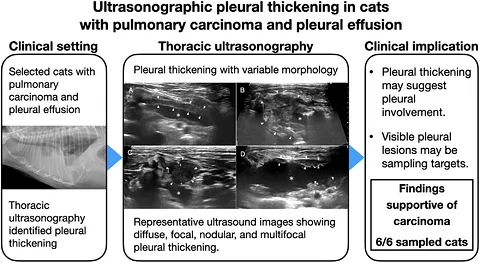

**Abbreviations**
CT: computed tomography
FNA: fine‐needle aspiration
IQR: interquartile range
SD: standard deviation

## 1 Introduction

Feline primary pulmonary carcinoma is an uncommon malignant epithelial neoplasm, accounting for approximately 0.69%–0.75% of diagnoses in feline postmortem examinations (Santos et al. 2023). These necropsy‐based estimates should not be interpreted as antemortem clinical prevalence, but they highlight the relative infrequency of this neoplasm in cats. Affected cats are typically older, and prognosis is often poor because metastatic disease is common at the time of diagnosis or develops during the disease course (Santos et al. 2023; Nunley et al. 2015).

Feline pulmonary carcinoma has a distinctive and clinically important metastatic pattern. Reported metastatic sites include the digits, skin, skeletal muscle, ocular tissues, bone, regional lymph nodes, pleura, and abdominal organs (Santos et al. 2023; Nunley et al. 2015; Ambrosini et al. 2018; Binanti and Zani 2015; Cassotis et al. 1999; Favrot and Degorce‐Rubiales 2005; Gionfriddo et al. 1990; Goldfinch and Argyle 2012; Gottfried et al. 2000; Masuyama et al. 2024; Nakanishi et al. 2003; Petterino et al. 2005; Sugiyama et al. 2010; Thrift et al. 2017; van der Linde‐Sipman and van den Ingh 2000). Digital metastasis is well recognised as part of the so‐called feline lung‐digit syndrome, but intrathoracic metastatic involvement is also clinically relevant (Santos et al. 2023; Nunley et al. 2015; Goldfinch and Argyle 2012; Thrift et al. 2017). Pleural involvement may be associated with pleural effusion, and pleural effusion has been reported as an adverse clinical finding in cats with primary pulmonary neoplasia (Nunley et al. 2015). Recognition of pleural disease may therefore be important for staging, prognosis, and selection of diagnostic sampling sites.

Diagnostic imaging plays a central role in the evaluation of cats with suspected pulmonary carcinoma (Nunley et al. 2015; Aarsvold et al. 2015; Forrest and Graybush 1998). Thoracic radiography is commonly used to identify pulmonary masses and metastatic pulmonary patterns, whereas computed tomography provides more complete thoracic staging and can identify findings such as pleural fluid, pleural thickening, lymphadenopathy, and extrapulmonary involvement (Nunley et al. 2015; Aarsvold et al. 2015; Forrest and Graybush 1998). Computed tomography studies have therefore already demonstrated that pleural abnormalities can be detected clinically in cats with pulmonary neoplasia, not only at necropsy (Nunley et al. 2015; Aarsvold et al. 2015). However, CT may not be available in all clinical settings, and some cats with respiratory compromise may initially undergo thoracic ultrasonography as part of stabilisation, thoracocentesis planning, or focused thoracic assessment.

Thoracic ultrasonography, including point‐of‐care and focused thoracic ultrasound techniques, is increasingly used in small animal practice to detect pleural and pericardial effusion and to support clinical decision‐making in unstable patients (Lisciandro 2011; Lisciandro 2021). In cats with pleural effusion, fluid may create an acoustic window that permits evaluation of the parietal and visceral pleural surfaces. This creates an opportunity to identify pleural thickening or pleura‐associated masses that may not be apparent on survey radiographs. In addition, ultrasound‐guided fine‐needle aspiration is an established technique for sampling peripheral pulmonary lesions in dogs and cats (Wood et al. 1998). When a pulmonary mass is centrally located or surrounded by aerated lung, direct ultrasonographic access to the primary mass may be limited. In such cases, visible pleural or pleura‐associated lesions may provide alternative sampling targets.

Despite the clinical relevance of pleural involvement in feline pulmonary carcinoma, the thoracic ultrasonographic appearance of pleural abnormalities in affected cats has not been well characterised. Previous studies have described metastatic patterns, survival, and CT findings of feline pulmonary neoplasia, but detailed ultrasonographic descriptions of pleural thickening and associated cytologic sampling findings remain limited (Santos et al. 2023; Nunley et al. 2015; Aarsvold et al. 2015). Such information could help clinicians recognise suspicious pleural abnormalities during thoracic ultrasound examinations and determine whether lesion‐directed sampling should be considered.

The primary objective of this retrospective descriptive study was to characterise the ultrasonographic features of pleural thickening in cats with pulmonary carcinoma or metastatic epithelial neoplasia consistent with pulmonary origin and concurrent pleural effusion. The secondary objective was to summarise cytologic findings from pleural effusion and ultrasound‐guided sampling of thickened pleura or pleura‐associated lesions when performed. We hypothesised that pleural thickening would show recognisable ultrasonographic features and that thickened pleura or pleura‐associated lesions could serve as feasible sampling targets in selected cats.

## 2 Materials and Methods

### 2.1 Study Design and Setting

This retrospective descriptive study was performed at Japan Small Animal Medical Center, Saitama, Japan. Medical records and archived thoracic ultrasound images of client‐owned cats evaluated between September 2011 and February 2023 were reviewed. The study was designed to describe ultrasonographic features of pleural thickening in a selected cohort of cats with pulmonary carcinoma and pleural effusion, rather than to estimate the prevalence, complete anatomic distribution, sensitivity, or specificity of thoracic ultrasonography for pleural metastatic disease.

### 2.2 Case Selection

Cats were eligible for inclusion if they met the following criteria: (1) pleural effusion evaluated by thoracic ultrasonography; (2) cytologic or histopathologic diagnosis of pulmonary carcinoma, or cytologic or histopathologic diagnosis of metastatic epithelial malignant neoplasia at a metastatic site in conjunction with imaging findings consistent with a primary pulmonary neoplasm; and (3) available archived thoracic ultrasound images permitting review of the pleural surface.

For the purpose of describing diagnostic certainty, cats were categorised according to the site from which neoplasia was confirmed. Cats were classified as having confirmed pulmonary carcinoma when cytology or histopathology was obtained from a pulmonary mass. Cats were classified as having presumptive pulmonary carcinoma when epithelial malignant neoplasia was confirmed from pleural effusion, pleura, a pleura‐associated thoracic wall lesion, or another metastatic site and thoracic imaging supported a primary pulmonary neoplasm. The sampled site or sites, diagnostic method, and cytologic or histopathologic diagnosis were recorded for each cat.

Available medical record data included signalment, body weight, presenting clinical signs, presence of respiratory signs, thoracic radiographic findings, thoracic computed tomographic findings when available, ultrasound findings, sites sampled for cytology or histopathology, and cytologic or histopathologic diagnoses.

### 2.3 Thoracic Ultrasonography

Thoracic ultrasonography was performed during routine clinical care by a veterinarian who had 15 years of experience in diagnostic imaging as of the end of the study period. Cats were positioned in right or left lateral recumbency without sedation. Ultrasound examinations were performed using linear transducers with frequencies up to 18 MHz or microconvex transducers with frequencies up to 9 MHz on 1 of 3 ultrasound systems: HI VISION Avius (Hitachi Medical Corporation, Tokyo, Japan) in 2 cats, ARIETTA 70 (Hitachi Aloka Medical, Ltd., Tokyo, Japan) in 10 cats, or ARIETTA 850 (FUJIFILM Healthcare Corporation, Chiba, Japan) in 10 cats. Cine clips were available for 20 cats. All included examinations had archived images considered adequate for review of the prespecified ultrasonographic variables; however, image quality was not formally scored or compared among ultrasound systems.

Because examinations were performed for clinical purposes, the pleural scanning protocol was not standardised for research. Pleural scanning was initially focused on the cranial pleural region. When cranial pleural thickening was not identified, the middle and caudal pleural regions were also scanned. Therefore, recorded pleural location and distribution were interpreted as descriptive findings based on the available images and were not considered to represent complete anatomic mapping of the thoracic pleura.

### 2.4 Image Review

Archived thoracic ultrasound still images and cine clips, when available, were reviewed in consensus by 3 observers: 2 board‐certified veterinary radiologists and 1 veterinarian who had 15 years of experience in diagnostic imaging as of the end of the study period. Images were reviewed using OsiriX MD software (Pixmeo, Geneva, Switzerland). The available image set for each cat was reviewed for the presence, location, distribution, maximal thickness, shape, echogenicity, homogeneity, and suspected intercostal muscle invasion of pleural thickening.

Maximal pleural thickness was measured in millimetres at the thickest visible portion of the pleural abnormality, perpendicular to the pleural surface when possible. Location was recorded as cranial, middle, or caudal. The cranial pleural region was defined as cranial to the cardiac silhouette, the middle region as the level of the cardiac silhouette, and the caudal region as caudal to the cardiac silhouette.

Distribution was categorised according to the available stored images. Focal pleural thickening was defined as a single localised area of thickening. Multifocal pleural thickening was defined as 2 or more spatially separated areas of thickening visible on the available images. Diffuse pleural thickening was defined as continuous thickening extending along the imaged pleural surface within the available image set. These categories were used as image‐based descriptors and did not imply complete assessment of the entire thoracic pleural surface.

Shape was categorised as smooth, irregular, or nodular according to the predominant ultrasonographic appearance. Smooth thickening was defined as broad‐based pleural thickening with a relatively even luminal margin. Irregular thickening was defined as pleural thickening with an uneven, angulated, or lobulated margin. Nodular thickening was defined as a discrete, mass‐like pleural protrusion arising from or attached to the pleural surface. When more than one shape was present, the predominant appearance was recorded.

Echogenicity was assessed relative to the adjacent intercostal muscle and recorded as hypoechoic, isoechoic, hyperechoic, or mixed. Mixed echogenicity was recorded when more than one echogenicity was present within the same pleural lesion. Homogeneity was recorded separately as homogeneous or heterogeneous according to whether the internal echotexture was uniform or nonuniform. Suspected intercostal muscle invasion was recorded when pleural thickening had an ill‐defined or irregular interface with the adjacent intercostal muscle, with apparent extension into the intercostal musculature or loss of the normal intervening fascial plane.

### 2.5 Cytology and Histopathology

Cytologic and histopathologic results were retrieved from the medical records. The sampled site, sampling method, and diagnosis were recorded for pulmonary masses, pleural effusion, thickened pleura, pleura‐associated thoracic wall lesions, and other metastatic sites when available. For ultrasound‐guided sampling of thickened pleura or pleura‐associated lesions, available procedural details, including needle gauge, number of passes, aspiration technique, and cytology or histopathology interpretation, were recorded when available.

### 2.6 Statistical Analysis

Data were summarised descriptively. Categorical variables were reported as counts and percentages, with denominators adjusted according to data availability. Continuous variables were summarised as mean ± standard deviation and range; median and interquartile range were also reported because of the small sample size. No imputation was performed for missing data. No formal hypothesis testing, confidence interval estimation, diagnostic accuracy analysis, sensitivity analysis, or specificity analysis was performed because the study lacked systematic pleural screening, an independent reference standard for pleural metastasis in all cats, and a non‐neoplastic comparison group. Statistical summaries were generated using R, version 4.4.3 (R Foundation for Statistical Computing, Vienna, Austria).

### 2.7 Ethical Approval

Ethical review and approval were not sought for this retrospective study because all clinical data and diagnostic images were obtained from client‐owned cats during routine clinical care at Japan Small Animal Medical Center, Saitama, Japan, and no additional procedures were performed for research purposes. Owners provided consent for future research use of clinical information, including diagnostic imaging data.

## 3 Results

### 3.1 Study Population and Clinical Findings

Twenty‐two cats met the inclusion criteria. The mean age was 11.8 ± 2.8 years, with a median age of 11 years (interquartile range [IQR], 10–14 years; range, 8–16 years). Mean body weight was 3.7 ± 0.9 kg, with a median body weight of 3.6 kg (IQR, 3.1–4.3 kg; range, 2.0–5.3 kg). There were 14 spayed females, 7 neutered males, and 1 sexually intact male. Breeds included domestic shorthair cats in 15 cases, American Shorthair cats in 5 cases, 1 Abyssinian, and 1 Munchkin. Respiratory clinical signs, defined as tachypnea, cough, or exercise intolerance, were recorded in 16/22 cats (72.7%). Tachypnea was recorded in 12 cats, cough in 3 cats, and exercise intolerance in 1 cat. Other presenting complaints included decreased appetite or activity in 12 cats, vomiting in 3 cats, weight loss in 2 cats, forelimb lameness in 1 cat, and intermittent diarrhoea in 1 cat.

Thoracic radiography and thoracic ultrasonography were performed in all 22 cats. Thoracic computed tomography was performed in 9/22 cats (40.9%). Pleural effusion was present in all cats and was recorded as minimal in 1 cat, small in 12 cats, moderate in 7 cats, and large in 2 cats. Pleural effusion was described as non‐turbid in 19 cats and turbid in 3 cats.

Clinical and diagnostic characteristics are summarised in Table 1.

**TABLE 1:** Clinical and diagnostic characteristics of 22 cats with pulmonary carcinoma or metastatic epithelial neoplasia consistent with pulmonary origin and pleural effusion.

Characteristic	Result
Number of cats	22
Signalment	
Age, years, mean ± SD	11.8 ± 2.8
Age, years, median (IQR)	11 (10–14)
Age, years, range	8–16
Body weight, kg, mean ± SD	3.7 ± 0.9
Body weight, kg, median (IQR)	3.6 (3.1–4.3)
Body weight, kg, range	2.0–5.3
Spayed female	14/22 (63.6%)
Neutered male	7/22 (31.8%)
Sexually intact male	1/22 (4.5%)
Domestic shorthair	15/22 (68.2%)
American Shorthair	5/22 (22.7%)
Abyssinian	1/22 (4.5%)
Munchkin	1/22 (4.5%)
Clinical signs	
Respiratory signs	16/22 (72.7%)
Tachypnea	12/22 (54.5%)
Cough	3/22 (13.6%)
Exercise intolerance	1/22 (4.5%)
Decreased appetite or activity	12/22 (54.5%)
Vomiting	3/22 (13.6%)
Weight loss	2/22 (9.1%)
Forelimb lameness	1/22 (4.5%)
Intermittent diarrhoea	1/22 (4.5%)
Imaging performed	
Thoracic radiography	22/22 (100%)
Thoracic ultrasonography	22/22 (100%)
Thoracic computed tomography	9/22 (40.9%)
HI VISION Avius	2/22 (9.1%)
ARIETTA 70	10/22 (45.5%)
ARIETTA 850	10/22 (45.5%)
Cine clips available	20/22 (90.9%)
Pleural effusion	
Pleural effusion present	22/22 (100%)
Minimal pleural effusion	1/22 (4.5%)
Small pleural effusion	12/22 (54.5%)
Moderate pleural effusion	7/22 (31.8%)
Large pleural effusion	2/22 (9.1%)
Non‐turbid pleural effusion	19/22 (86.4%)
Turbid pleural effusion	3/22 (13.6%)
Diagnostic confirmation	
Pulmonary mass FNA performed	12/22 (54.5%)
Pulmonary mass FNA diagnostic for carcinoma or malignant epithelial neoplasia	12/12 (100%)
Presumptive pulmonary carcinoma without pulmonary mass cytology	10/22 (45.5%)
Diagnostic support from pleural effusion cytology or cell block among presumptive cases	6/10 (60.0%)
Diagnostic support from pleural or pleura‐associated thoracic wall lesion sampling among presumptive cases	3/10 (30.0%)
Diagnostic support from soft tissue metastatic lesion cytology among presumptive cases	1/10 (10.0%)
Pleural effusion cytology performed	20/22 (90.9%)
Neoplastic cells identified in pleural effusion cytology	15/20 (75.0%)
No neoplastic cells identified in pleural effusion cytology	5/20 (25.0%)
Ultrasound‐guided sampling of thickened pleura or pleura‐associated lesion	6/22 (27.3%)
Malignant neoplasia diagnosed from sampled lesion	6/6 (100%)

*Note*: Data are presented as number/total number (%) unless otherwise specified. Percentages were rounded to one decimal place. FNA, fine‐needle aspiration; IQR, interquartile range; SD, standard deviation. Presumptive pulmonary carcinoma indicates cats without pulmonary mass cytology that had thoracic imaging findings consistent with a primary pulmonary neoplasm and cytologic or histopathologic evidence of malignant epithelial neoplasia from pleural effusion, pleura, a pleura‐associated thoracic wall lesion, or another metastatic site.

### 3.2 Diagnostic Confirmation

Pulmonary mass FNA was performed in 12/22 cats and yielded cytologic diagnoses consistent with pulmonary carcinoma or malignant epithelial neoplasia in all 12. The remaining 10 cats did not have cytologic confirmation from the pulmonary mass and were included based on thoracic imaging findings consistent with a primary pulmonary neoplasm and cytologic or histopathologic evidence of malignant neoplasia from pleural effusion, thickened pleura or a pleura‐associated thoracic wall lesion, or another metastatic site. In these 10 cats, diagnostic support was obtained from pleural effusion cytology or pleural effusion cell block in 6 cats, pleural or pleura‐associated thoracic wall lesion sampling in 3 cats, and cytology of soft tissue metastatic lesions in 1 cat.

Pleural effusion cytology was performed in 20/22 cats. Neoplastic cells were identified in pleural effusion cytology in 15/20 cats (75.0%). No neoplastic cells were identified in pleural effusion cytology in five cats.

Ultrasound‐guided sampling of thickened pleura or a pleura‐associated thoracic wall lesion was performed in six cats; the final cytologic or histopathologic findings were supportive of carcinoma in all six. Five samples were interpreted as epithelial malignant neoplasia. In one cat, ultrasound‐guided FNA was initially interpreted as suspicious for non‐epithelial malignant neoplasia, and subsequent biopsy of a thoracic wall mass confirmed squamous cell carcinoma. Of the 6 sampled lesions, 4 were classified ultrasonographically as diffuse and 2 as multifocal. Five were irregular and one was smooth. No nodular pleural lesions were sampled.

### 3.3 Ultrasonographic Findings

Thoracic ultrasonography was performed using HI VISION Avius in 2/22 cats (9.1%), ARIETTA 70 in 10/22 cats (45.5%), and ARIETTA 850 in 10/22 cats (45.5%). Cine clips were available for 20/22 cats (90.9%).

Pleural thickening was recorded in all 22 cats. Based on the available stored ultrasound images, pleural thickening was classified as diffuse in 12/22 cats (54.5%), focal in 6/22 cats (27.3%), and multifocal in 4/22 cats (18.2%). Because pleural scanning was not performed systematically across all thoracic regions, these categories were interpreted as descriptive image‐based findings rather than complete anatomic mapping of pleural disease.

The representative or measured pleural abnormality was located in the cranial pleural region in 18 cats, the middle pleural region in 3 cats, and the caudal pleural region in one cat. Maximal visible pleural thickness was 5.6 ± 3.3 mm, with a median of 4.9 mm (IQR, 3.3–6.6 mm; range, 1.2–16.2 mm).

Pleural thickening was smooth in 9/22 cats (40.9%), irregular in 8/22 cats (36.4%), and nodular in 5/22 cats (22.7%). Echogenicity relative to the adjacent intercostal muscle was hypoechoic in 15 cats, isoechoic in 2 cats, mixed isoechoic and hypoechoic in 4 cats, and mixed hyperechoic and hypoechoic in 1 cat. Internal echotexture was homogeneous in 14 cats and heterogeneous in 8 cats. Homogeneous lesions included 12 hypoechoic and 2 isoechoic lesions. Heterogeneous lesions included 4 lesions with mixed isoechoic and hypoechoic echogenicity, 3 hypoechoic lesions, and 1 lesion with mixed hyperechoic and hypoechoic echogenicity.

Ultrasonographic features of pleural thickening are summarised in Table 2, and representative images are shown in Figure 1.

**TABLE 2:** Ultrasonographic features of pleural thickening in 22 cats with pulmonary carcinoma or metastatic epithelial neoplasia consistent with pulmonary origin and pleural effusion.

Ultrasonographic feature	Result
Pleural thickening identified	22/22 (100%)
Distribution based on available stored images	
Diffuse thickening	12/22 (54.5%)
Focal thickening	6/22 (27.3%)
Multifocal thickening	4/22 (18.2%)
Region of representative or measured pleural abnormality	
Cranial pleural region	18/22 (81.8%)
Middle pleural region	3/22 (13.6%)
Caudal pleural region	1/22 (4.5%)
Maximal visible pleural thickness	
Maximal visible pleural thickness, mm, mean ± SD	5.6 ± 3.3
Maximal visible pleural thickness, mm, median (IQR)	4.9 (3.3–6.6)
Maximal visible pleural thickness, mm, range	1.2–16.2
Shape	
Smooth	9/22 (40.9%)
Irregular	8/22 (36.4%)
Nodular	5/22 (22.7%)
Echogenicity relative to adjacent intercostal muscle	
Hypoechoic	15/22 (68.2%)
Isoechoic	2/22 (9.1%)
Mixed isoechoic and hypoechoic	4/22 (18.2%)
Mixed hyperechoic and hypoechoic	1/22 (4.5%)
Internal echotexture	
Homogeneous	14/22 (63.6%)
Heterogeneous	8/22 (36.4%)
Suspected intercostal muscle invasion	
Suspected intercostal muscle invasion	8/22 (36.4%)
Irregular shape among lesions with suspected intercostal muscle invasion	5/8 (62.5%)
Nodular shape among lesions with suspected intercostal muscle invasion	3/8 (37.5%)
Smooth shape among lesions with suspected intercostal muscle invasion	0/8 (0%)
Maximal thickness of lesions with suspected intercostal muscle invasion, mm, median (IQR)	7.1 (5.6–8.5)
Maximal thickness of lesions with suspected intercostal muscle invasion, mm, range	4.3–16.2
Ultrasound‐guided lesion sampling	
Ultrasound‐guided lesion sampling performed	6/22 (27.3%)
Diffuse sampled lesion	4/6 (66.7%)
Multifocal sampled lesion	2/6 (33.3%)
Focal sampled lesion	0/6 (0%)
Irregular sampled lesion	5/6 (83.3%)
Smooth sampled lesion	1/6 (16.7%)
Nodular sampled lesion	0/6 (0%)
Final cytologic or histopathologic findings supportive of carcinoma	6/6 (100%)

*Note*: Data are presented as number/total number (%) unless otherwise specified. Percentages were rounded to one decimal place. Distribution and location were recorded from available stored images and should not be interpreted as complete anatomic mapping of the thoracic pleura because the clinical scanning protocol was not standardised across all thoracic regions. Mixed echogenicity indicates more than one echogenicity within the same lesion; internal echotexture was recorded separately as homogeneous or heterogeneous. Suspected intercostal muscle invasion was based on ultrasonographic appearance and was not histologically confirmed in all cats. Final cytologic or histopathologic findings supportive of carcinoma include one cat in which ultrasound‐guided FNA was initially suspicious for non‐epithelial malignant neoplasia and subsequent biopsy of a thoracic wall mass confirmed squamous cell carcinoma.

**FIGURE 1:**
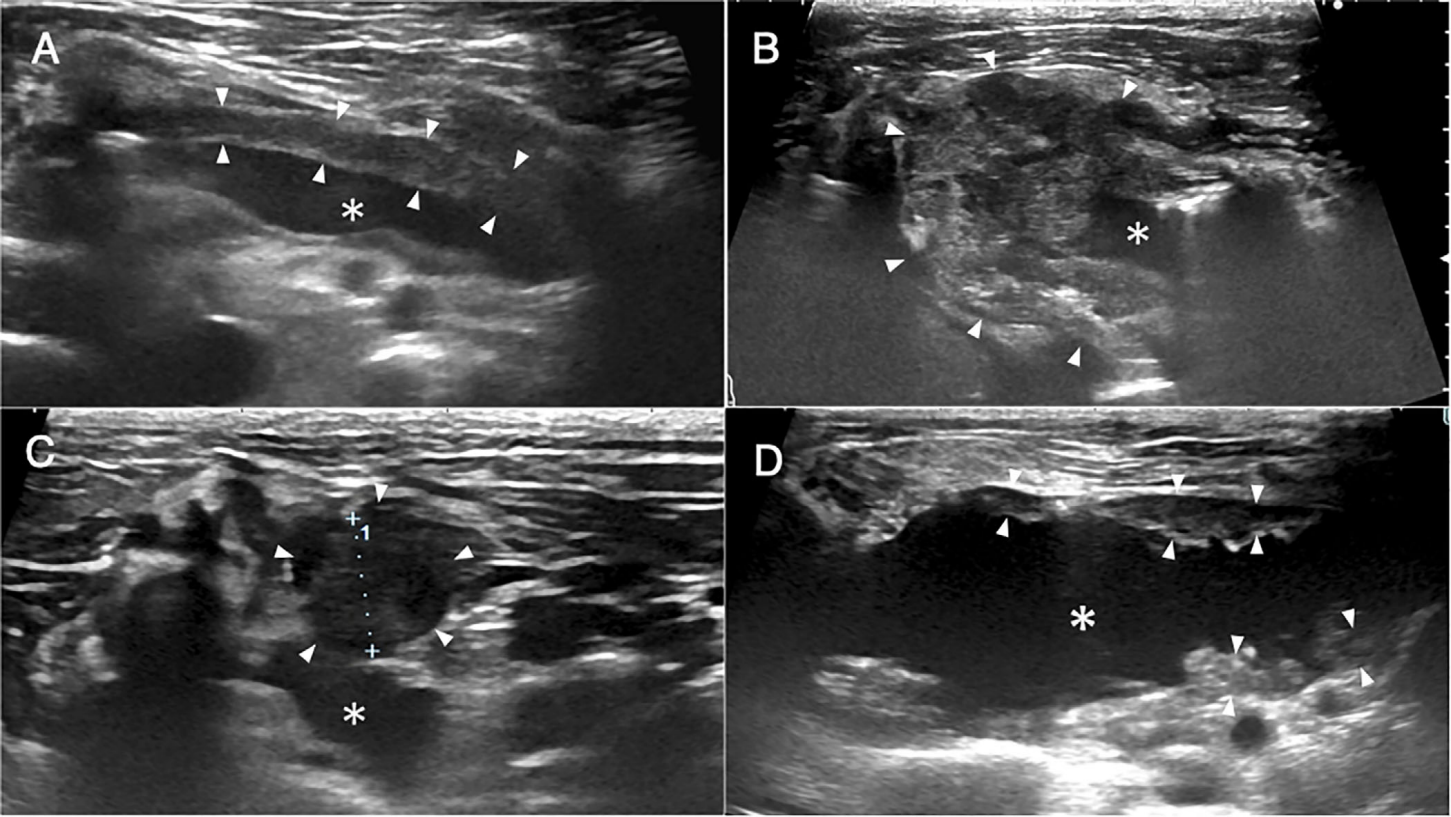
Representative thoracic ultrasonographic images of pleural thickening in cats with pulmonary carcinoma or metastatic epithelial neoplasia consistent with pulmonary origin and pleural effusion. Images were obtained using a linear transducer. The asterisk indicates pleural effusion, and arrowheads indicate pleural thickening. (A) Diffuse, homogeneously hypoechoic pleural thickening with a smooth margin. (B) Focal pleural thickening at the cranial pleural reflection with irregular margins and mixed hyperechoic and hypoechoic echogenicity. This was the thickest pleural lesion recorded in the study. (C) Focal, nodular, homogeneously hypoechoic pleural thickening. The arrow indicates distal acoustic shadowing from costal cartilage. (D) Multifocal pleural thickening with irregular margins, heterogeneous echotexture, and overall hypoechogenicity.

Suspected intercostal muscle invasion was recorded in 8/22 cats (36.4%). Of these 8 lesions, 5 were irregular and three were nodular; no smooth lesions were classified as having suspected intercostal muscle invasion. The median maximal thickness of lesions with suspected intercostal muscle invasion was 7.1 mm (IQR, 5.6–8.5 mm; range, 4.3–16.2 mm).

A representative example of suspected intercostal muscle invasion is shown in Figure 2.

**FIGURE 2:**
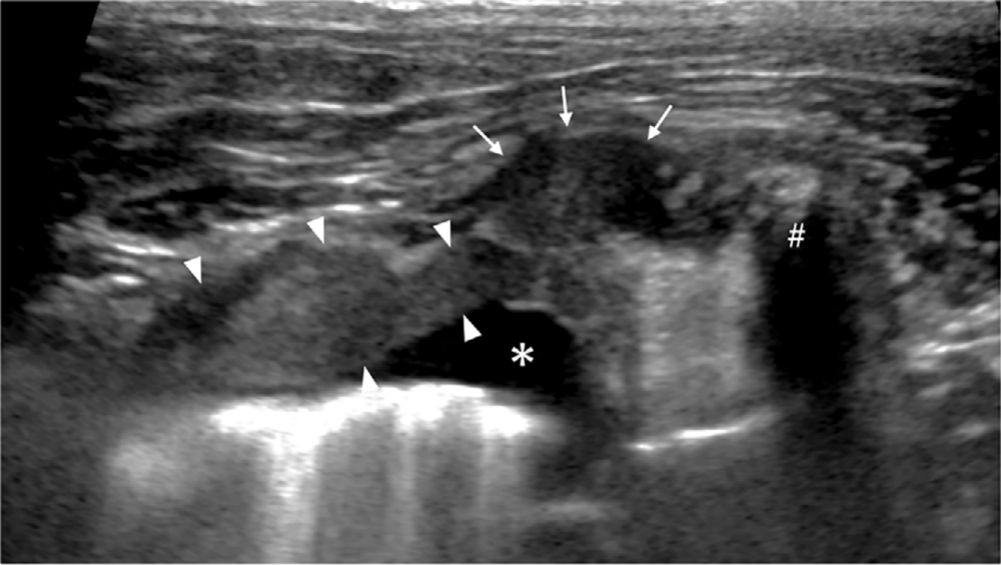
Thoracic ultrasonographic image of pleural thickening with suspected intercostal muscle invasion in a cat with pulmonary carcinoma and pleural effusion. Hypoechoic pleural thickening (arrowheads) has an irregular interface with the adjacent intercostal musculature and appears to extend into the intercostal muscle region (arrows). The asterisk indicates pleural effusion, and the number sign indicates a rib with distal acoustic shadowing. This appearance was recorded as suspected intercostal muscle invasion; histologic confirmation of intercostal muscle invasion was not available in all cats.

## 4 Discussion

This retrospective descriptive study documented thoracic ultrasonographic features of pleural thickening in a selected cohort of 22 cats with pulmonary carcinoma or metastatic epithelial neoplasia consistent with pulmonary origin and concurrent pleural effusion. Pleural thickening had variable morphology and echotexture, and suspected intercostal muscle invasion was recorded in a subset of cats. The principal finding was therefore the range of ultrasonographic appearances of pleural thickening in this clinical population. A secondary, clinically relevant observation was that thickened pleura or pleura‐associated lesions provided an accessible sampling target in six selected cats. Because inclusion required pleural effusion and available thoracic ultrasound images and the pleura was not scanned systematically, these findings should not be interpreted as estimates of prevalence, regional predilection, or diagnostic accuracy.

Pleural involvement is a recognised manifestation of feline pulmonary carcinoma, particularly in advanced disease. Necropsy and computed tomographic studies have documented pleural metastasis, pleural effusion, and pleural thickening in affected cats (Santos et al. 2023; Nunley et al. 2015; Aarsvold et al. 2015). In the present cohort, pleural thickening was visible on the reviewed ultrasound images in all cases. This observation does not indicate high sensitivity of thoracic ultrasonography for pleural metastatic disease, because the study did not include cats without pleural effusion, lacked systematic whole‐pleura scanning, and did not require cytologic or histopathologic confirmation of each pleural lesion. Instead, it indicates that the pleural surface merits deliberate assessment when thoracic ultrasonography is performed in a cat with suspected pulmonary carcinoma and pleural effusion.

Pleural effusion likely facilitated visualisation by separating the lung from the thoracic wall and providing an acoustic window to pleural and peripheral thoracic structures (Lisciandro 2011; Lisciandro 2021; Reichle and Wisner 2000). Thoracic point‐of‐care ultrasonography is used to detect pleural fluid and support urgent clinical decision‐making (Lisciandro 2011; Lisciandro 2021), and conventional noncardiac thoracic ultrasonography can aid evaluation and image‐guided sampling of accessible intrathoracic lesions in dogs and cats (Reichle and Wisner 2000). In the present cohort, respiratory clinical signs were recorded in 16 of 22 cats, and all cats underwent thoracic radiography and ultrasonography. Therefore, the study population likely represents cats with clinically apparent thoracic disease rather than the broader population of cats with pulmonary carcinoma. This selection should be considered when applying the results to cats without pleural effusion or cats with earlier‐stage pulmonary carcinoma.

The ultrasonographic appearance of pleural thickening was variable. Smooth thickening was the most frequent shape, followed by irregular and nodular thickening. Most lesions were hypoechoic relative to the adjacent intercostal muscle, although mixed echogenicity was also observed. Previous veterinary imaging studies have also described varied pleural abnormalities in malignant pleural disease and pleural carcinomatosis (Reetz et al. 2019; Weston et al. 2021). However, pleural morphology alone is unlikely to be specific. Computed tomographic studies of dogs and cats with pleural effusion found that nodular pleural thickening and pleural masses were associated with malignancy but also reported substantial overlap among malignant and nonmalignant effusion categories (Reetz et al. 2019; Watton et al. 2017). Although these studies used computed tomography or included different disease populations, they support cautious interpretation of the current ultrasonographic findings. Therefore, ultrasonographic pleural thickening in this setting should be considered suspicious for pleural involvement, especially when accompanied by malignant pleural effusion or carcinoma at another site, but should not be considered diagnostic without cytologic or histopathologic confirmation.

Suspected intercostal muscle invasion was recorded in 8 cats and was confined to lesions with irregular or nodular morphology. In a canine computed tomographic study, signs of thoracic wall invasion were observed only in dogs with malignant pleural effusion, although substantial overlap in other pleural features remained (Watton et al. 2017). This comparison is supportive but indirect because the previous study involved dogs and computed tomography, whereas the present classification was based on ultrasonography in cats. Moreover, histopathologic confirmation of muscle invasion was unavailable in most cats. The term suspected intercostal muscle invasion is therefore appropriate, and the imaging criterion used here should be regarded as a descriptive feature rather than a validated marker of true invasion. Prospective comparison with computed tomography, surgery, necropsy, or histopathology is needed before this finding can be used for staging or prognostication.

Ultrasound‐guided sampling of thickened pleura or pleura‐associated lesions yielded findings supportive of carcinoma in all six sampled cats. Previous veterinary studies have shown that ultrasonography can facilitate diagnostic sampling of peripheral pulmonary and other accessible noncardiac intrathoracic lesions in dogs and cats (Wood et al. 1998; Reichle and Wisner 2000). The present findings extend that practical concept to visible pleural abnormalities in selected cats with suspected pulmonary carcinoma. Such lesions may provide an alternative sampling target when the primary pulmonary mass is centrally located, separated from the thoracic wall by aerated lung, or otherwise considered less accessible. Nevertheless, only six cats underwent lesion sampling, selection was based on clinical judgment and accessibility, and detailed procedural variables were inconsistently recorded. These results therefore support feasibility in selected cats but do not establish diagnostic accuracy, safety, or superiority over pulmonary mass aspiration or other sampling methods.

Pleural effusion cytology identified neoplastic cells in 15 of 20 cats in this cohort. No neoplastic cells were identified in the remaining 5 cats despite other evidence supporting pulmonary carcinoma or metastatic epithelial neoplasia. Thus, within this selected cohort, a negative pleural fluid cytology result did not exclude carcinoma. Direct sampling of a visible pleural or pleura‐associated lesion may be considered when pleural effusion cytology is negative or equivocal and a safe ultrasonographic sampling window is present. This clinical implication remains hypothesis‐generating because lesion sampling was performed in a small, nonrandom subset and was not compared prospectively with pleural fluid cytology or pulmonary mass aspiration.

This study has several limitations. First, the retrospective design introduced selection bias. Cats were identified from cases that underwent thoracic ultrasonography and had pleural effusion, which likely enriched the cohort for cats with clinically apparent or advanced thoracic disease. Therefore, the findings should not be generalised to all cats with pulmonary carcinoma, particularly cats without pleural effusion or cats with earlier‐stage disease.

Second, diagnostic certainty varied among cats. Pulmonary carcinoma was confirmed by pulmonary mass cytology in 12 cats, whereas the remaining cats were included based on thoracic imaging findings consistent with a primary pulmonary neoplasm and cytologic or histopathologic evidence of malignant epithelial neoplasia from pleural effusion, pleura, pleura‐associated thoracic wall lesions, or other metastatic sites. Although this approach reflects clinical practice in cats in which direct pulmonary mass sampling is not always feasible, an occult extrapulmonary primary tumour could not be completely excluded in all presumptive cases.

Third, the thoracic ultrasound protocol was not standardised. The cranial pleural region was assessed first, and middle and caudal pleural regions were scanned only when cranial pleural thickening was not identified. Consequently, this study cannot determine the true number of pleural lesions, complete anatomic distribution, or regional predilection of pleural abnormalities. The recorded distribution and location should therefore be interpreted as descriptive findings based on the available stored images rather than complete anatomic mapping of the thoracic pleura.

Fourth, not all pleural lesions were cytologically or histologically confirmed. Some ultrasonographic abnormalities, particularly diffuse pleural thickening, may have represented reactive or inflammatory pleural changes rather than neoplastic infiltration. In addition, the study lacked a comparison group of cats with non‐neoplastic pleural effusion, such as inflammatory, chylous, or cardiogenic effusion; therefore, specificity of the ultrasonographic findings could not be assessed.

Fifth, image acquisition and review had inherent limitations. Ultrasound examinations were performed over a long study period using three ultrasound systems, image quality was not formally scored, and cine clips were unavailable in 2 cats. These factors may have introduced variability in image interpretation. In addition, images were reviewed by consensus, and interobserver agreement was not assessed; therefore, reproducibility of the ultrasonographic categories remains unknown.

Finally, the sample size was small, particularly for cats undergoing ultrasound‐guided sampling of thickened pleura or pleura‐associated lesions. The decision to sample a lesion was made during clinical care and likely depended on lesion accessibility, clinician judgment, and patient status. Detailed procedural variables for lesion sampling, including needle gauge, number of passes, and aspiration technique, were not consistently available in the medical records. Therefore, although the final cytologic or histopathologic findings were supportive of carcinoma in all sampled cats, this study cannot determine the diagnostic accuracy, sensitivity, specificity, or comparative value of pleural lesion sampling relative to pleural effusion cytology or pulmonary mass aspiration.

Prospective studies are warranted to evaluate thoracic ultrasonography in cats with pulmonary carcinoma using a standardised pleural scanning protocol that includes all thoracic regions regardless of whether cranial pleural abnormalities are identified. Such studies should include cats with and without pleural effusion, record the accessibility of pulmonary and pleural lesions for sampling, compare ultrasonographic findings with computed tomography and cytologic or histopathologic reference standards, and include non‐neoplastic pleural effusion controls when possible. These data would clarify the prevalence, distribution, diagnostic accuracy, and clinical significance of ultrasonographic pleural thickening in feline pulmonary carcinoma.

## 5 Conclusion

In this selected cohort of cats with pulmonary carcinoma or metastatic epithelial neoplasia consistent with pulmonary origin and concurrent pleural effusion, thoracic ultrasonography showed pleural thickening with variable morphology and echogenicity. Thickened pleura or pleura‐associated lesions provided feasible sampling targets in a small subset of cats. These findings support careful ultrasonographic assessment of the pleural surface in cats with pulmonary carcinoma and pleural effusion, while emphasising that prevalence, complete anatomic distribution, sensitivity, specificity, and prognostic significance require prospective evaluation.

## Author Contributions

**Atsushi Toshima**: conceptualisation, methodology, data curation, investigation, resources, visualisation, Writing – original draft. **Caroline V. Fulkerson**: methodology, investigation, writing – review & editing. **Masahiro Murakami**: conceptualisation, methodology, data curation, formal analysis, investigation, visualisation, project administration, supervision, writing – original draft, writing – review & editing. All authors reviewed and approved the final manuscript.

## Funding

The authors have nothing to report.

## Ethics Statement

Ethical review and approval were not sought for this retrospective study because all clinical data and diagnostic images were obtained from client‐owned cats during routine clinical care, and no additional procedures were performed for research purposes. Owners provided consent for future research use of clinical information, including diagnostic imaging data.

## Conflicts of Interest

The authors declare no conflicts of interest.

## Data Availability

The data that support the findings of this study are available from the corresponding author upon reasonable request, subject to institutional and client confidentiality restrictions.
